# What Are the Experiences of Mental Health Practitioners Involved in a Coroner’s Inquest and Other Inquiry Processes after an Unexpected Death of a Patient? A Systematic Review and Thematic Synthesis of the Literature

**DOI:** 10.3390/ijerph21030357

**Published:** 2024-03-18

**Authors:** Millie Tamworth, Sahra Tekin, Jo Billings, Helen Killaspy

**Affiliations:** Division of Psychiatry, University College London, London W1T 7NK, UK; sahra.tekin.20@ucl.ac.uk (S.T.); j.billings@ucl.ac.uk (J.B.); h.killaspy@ucl.ac.uk (H.K.)

**Keywords:** psychiatrists, mental health practitioners, suicide bereavement, homicide, qualitative research, vicarious trauma, occupational trauma, psychological support, systematic review

## Abstract

Grief after suicide or patient-perpetrated homicide can be complex for those involved in the patient’s care. Mental health practitioners with patients who die unexpectedly may be called to assist in the formal investigation processes that follow. The aim of this study was to examine the experience of mental health practitioners called to attend a coroner’s inquest or other forms of formal inquiry. A protocol for a systematic review was prospectively registered on PROSPERO (CRD42023400310). A thematic synthesis of existing literature was conducted. We identified six articles for inclusion and constructed three themes from our analysis: Blame and enduring hostility, In the dark, and Limited learning. We found mental health practitioners may construct narratives of self-blame. These can be reinforced by the investigatory processes that follow. Feedback from inquiries is often delivered haphazardly and may not reflect the realities of clinical work. The support given to assist practitioners through inquiry processes varied—both in amount and how helpful it was. The research conducted on this topic is limited. More qualitative research should be conducted to understand the factors that make this experience more or less difficult as well as well as what support is needed for whom.

## 1. Introduction

A coroner’s inquest is held after an unexpected death. An inquest is an inquisitorial process tasked with answering four specific questions in relation to a death: (1) who, (2) when, (3) where, and (4) how. It does not seek to assign civil or criminal liability. When deaths are complex, for instance, when an individual has died whilst in prison, in police custody, or whilst detained in hospital under the Mental Health Act, the parameters of the investigation are widened to consider the broader circumstances of the death. In these cases, the investigation is much more rigorous [1]. In England, when a suicide or homicide involves somebody under the care of mental health services, there are additional formal investigation processes conducted by the mental health organisation and, in many cases, an independent inquiry.

Practitioners involved in the care of a patient who dies whilst under the care of mental health services may be called to give evidence at the inquest and other local and independent inquiry processes. Multiple stakeholders may be involved in these proceedings. At the inquest, family members, legal representatives for the family, legal representatives for the mental health organisation, and, potentially, the media may all be present. Despite a non-adversarial remit, the inquest process can identify actions of individuals or organisations as having contributed to the death. Biddle [2] describes the inquest as ‘inevitably’ involving consideration of human agency and motivation. This can lead to individuals or organisations being identified as potentially accountable in some way for the death and can contribute to a culture of blame [3].

Research conducted on the impact of inquests on family members following a patient’s suicide suggests it can be unhelpful for the grieving process [2,4,5,6]. This is likely due to the multiple functions that the process has to deliver: it must recognise and potentially contain the grief of those mourning an unexpected bereavement [7], manage the raw and often conflicting perspectives of different stakeholders, and, at the same time, establish the circumstances of the death and make informed recommendations [5]. Sudden deaths such as suicide are complex, and grief is often intensified since there has been no opportunity for preparation for the death [8]. In addition, different types of sudden loss present different challenges. In the case of suicide and homicide, the involvement of the media and the legal system can further complicate the impact on the person’s loved ones [8]. Suicide has been found to be the most stigmatising of sudden losses [9], with that stigma manifesting itself in blame and shame [10], sometimes felt by those who are left behind. Perceptions of preventability may also generate guilt and difficult emotions, including anger and blame [8].

Patient suicide also impacts healthcare professionals; practitioners may be considered as ‘second victims’ following these kinds of adverse events [11]. In a previous study, interviews with consultant psychiatrists revealed that patient suicide was often associated with practitioners feeling blamed, guilty, and professionally isolated, and some suffered a period of poor mental health as a result [3]. Whilst there is growing awareness of the negative impact of patient suicide on practitioners [12,13,14,15,16,17], there has been less acknowledgement in the literature of the impact on practitioners of attending the coroner’s inquest and other parts of the inquiry process.

This systematic review aimed to report on the published evidence in relation to mental health practitioners’ experience of the coroner’s inquest and other inquiry processes following a patient suicide or patient-perpetrated homicide. Our specific objectives were to identify the factors which make the process more or less difficult as well as assess the type and effectiveness of any support received.

## 2. Methods

We followed the recommendations of the ‘Preferred Reporting Items for Systematic Reviews and Meta-Analyses’ (PRISMA) [18]. The study was registered in advance on the ‘Prospective Register or Systematic Reviews’ (PROSPERO)—CRD42023400310.

### 2.1. Search Strategy

Searches took place on the 3 February 2023 and were re-run in November 2023.

Eight databases were searched with no date restrictions: Medline, PsychINFO, EMBASE (all via Ovid), CINAHL Plus, IBSS, Pubmed, Web of Science and SciVerse Scopus. We also conducted a search across Dogpile, Google Advance and bibliographies for grey or unpublished literature. We used a combination of keywords and MeSH terms. The search was built around key search terms relating to three main concepts: (1) ‘mental health practitioner’, (2) ‘serious incident investigations, homicide inquiries, coroner inquest’, and (3) ‘experiences and expectations’. Search terms can be found in Supplementary File S1.

After retrieving articles, we transferred them to Endnote and removed any duplicates. Articles titles and abstracts were then screened for inclusion by MT with a second reviewer, ST, screening a randomly selected 20%. Full text versions of all articles selected for second screening were then independently screened by both ST and MT. Any disagreement on whether the publication met inclusion criteria was resolved through discussion.

### 2.2. Inclusion and Exclusion Criteria

Qualitative and quantitative studies were included providing the paper had sufficient extractable findings relating to mental health practitioners and their experience of a coroner’s inquest or other inquiry processes after a suicide or homicide. Systematic reviews, scoping and other types of literature review and non-empirical studies were excluded.

We defined ‘mental health practitioner’ as including anyone who is currently or has previously worked with patients of mental health services, including but not limited to psychiatrists, psychologists, nurses, occupational therapists, social workers, support workers and any other recognised clinical role in a multi-disciplinary mental health team. This included staff working for statutory (NHS and Local Authority) and non-statutory (voluntary sector) organisations. Studies reporting on staff who were not working in a clinical role were excluded, as were studies reporting on other stakeholders who may be present at an inquest (e.g., employees of coroner offices, police, or bereaved family members).

Papers focusing on patient safety events which did not result in the death of the patient by suicide or patient-perpetrated homicide were excluded, as were papers focusing on investigations involving non-mental health staff.

### 2.3. Data Extraction and Quality Appraisal

MT extracted key characteristics of included papers, as defined in the PROSPERO protocol, into a table. When data were not available, the authors were contacted. MT and ST independently conducted quality appraisals of the included papers. Many of the included studies were cross-sectional surveys. There is no specific quality assessment tool with established validity designed to assess cross-sectional surveys [19]. We, therefore, decided to use an adapted version of the Mixed Methods Appraisal Tool (MMAT) [20], a critical appraisal tool specifically designed for systematic reviews that include studies using mixed methods. Studies are ranked on a one-to-four-star system, with higher-quality papers given more stars. All studies were independently assessed by two researchers (MT and ST), and any differences of opinion were resolved through discussion. This is a relatively new research area, and our aim was to report on the calibre of existing research as well as its content. Therefore, studies were included in our analysis, irrespective of quality. We acknowledge the risk that this distorts the review’s findings or leads to incorrect conclusions [21]. We have sought to counter this risk by conducting quality assessments for each study and reflecting on these in our discussion.

### 2.4. Data Analysis

Six studies were relevant to our review questions. Our choice of a thematic synthesis approach reflected our review aims and the nature of available evidence [22]. Five of the six studies were surveys, often with limited qualitative data, making integrative synthesis challenging. Studies were uploaded to NVivo 14. Studies were read and re-read to facilitate immersion in the data [23]. All data directly referring to our research question on experiences of attending the inquest or other inquiry processes was extracted from the studies, including direct quotes and comments by the authors. We followed the three-staged approach outlined by Thomas and Harden [24]. (1) MT coded the original data with descriptive themes. (2) These were then combined and organised into related areas and discussed by the research team. (3) Finally, the research team assessed how the themes related to the review’s stated research questions and what generalisable themes were evident. Our thematic synthesis balanced our objectives of staying true to the experiences reported whilst also facilitating the production of new concepts and interpretations [24], which may be considered additive to the overall body of evidence already published [22].

### 2.5. Analytic Rigour

We followed ENTREQ guidelines [25] to maximise reliability by making our work replicable [26] and to allow readers to assess the dependability of findings [25]. The ENTREQ statement consists of 21 items grouped into five domains (introduction, method, methodology, literature search, appraisal and synthesis of findings) [25]. We used team meetings to develop our conceptual understanding of participant experiences and ensure the validity of our themes [26]. We explored our own reflexivity within the team by reflecting on personal experience as healthcare professionals (HK, JB) and our academic interests in the subject (MT, ST, JB, HK).

## 3. Results

### 3.1. Study Characteristics

**Figure 1:**
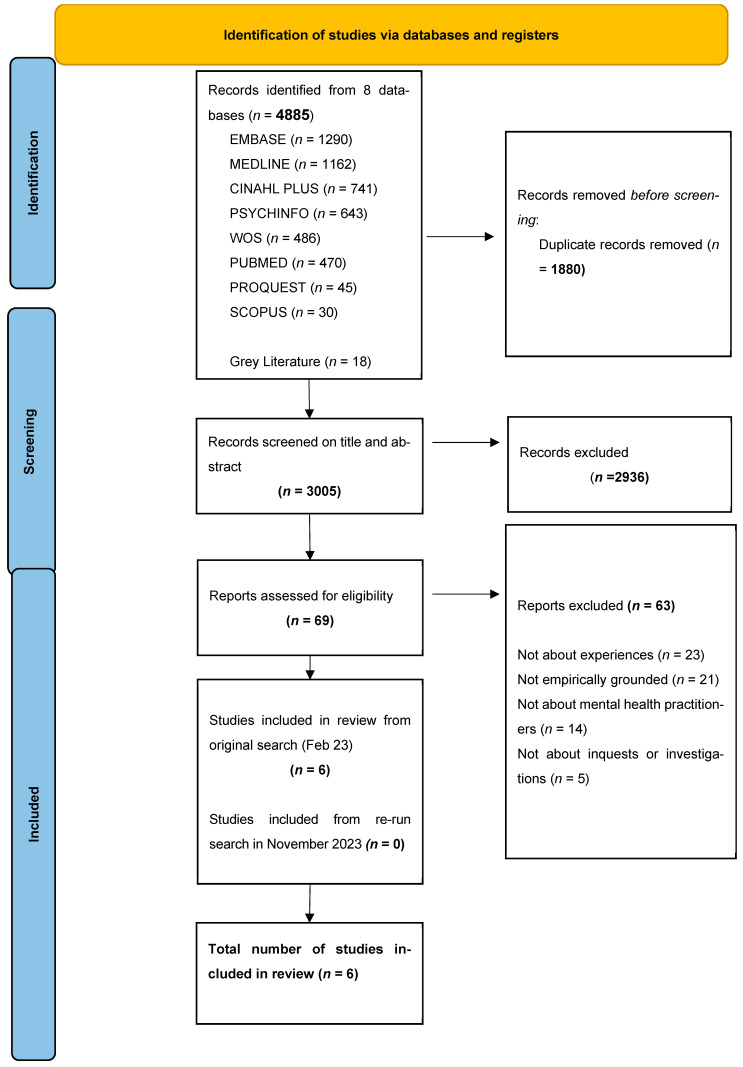
PRISMA flowchart.

A total of 4885 studies were retrieved from databases and an additional 18 from grey literature methods. After de-duplication, 3005 papers remained. After the title and abstracts were screened, 69 papers were retained and read in full by the reviewers. Following this, six studies were included for the thematic synthesis. Follow-up searches conducted in November 2023 did not contain any additional papers for inclusion. A PRISMA flowchart is shown in Figure 1.

Characteristics of included studies are shown in Table 1. Of the six studies identified, five were conducted in the UK (one in Scotland) and one in New Zealand. Five of the six studies had been completed within the past five years (since 2019) and one in 2000. Four of the six studies followed the same survey design, with three produced by the same organisation, the Royal College of Psychiatrists. Three studies focused on the experience of patient-perpetrated homicide, one of those specifically on the inquiry process. Three studies focused on patient suicide. The number of participants per study ranged from 10 to 167 (median, 137), and the studies together included a total of 642 participants. Of these, 502 were psychiatrists, 63 were nurses, 37 were psychologists or psychotherapists, 17 were support workers, and 15 were social workers. Data on sex was available for 594 participants, of whom 265 (45%) were male. Four studies were surveys that combined descriptive analytics and free text responses; one survey also included follow-up interviews, and one study used semi-structured interviews. The key findings of the study that relate to our research objectives are shown in Table 2.

**Figure 2:**
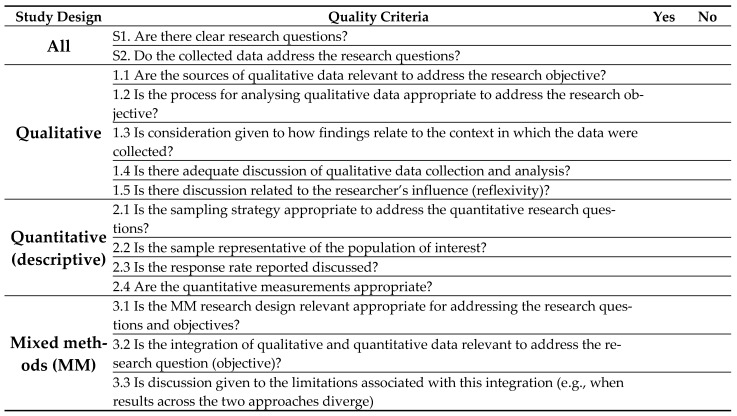
Mixed Methods Appraisal Tool [20].

The quality assessment tool [20] is shown in Figure 2. The results of the quality assessment are shown in Table 3. Five of the studies were rated as three stars and one study as two stars.

### 3.2. Thematic Synthesis

**Figure 3:**
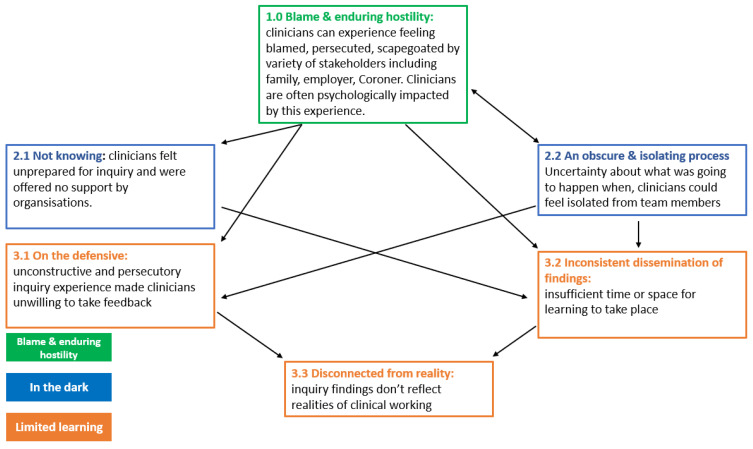
The interrelationship of different aspects of the practitioner’s experience.

**Figure 4:**
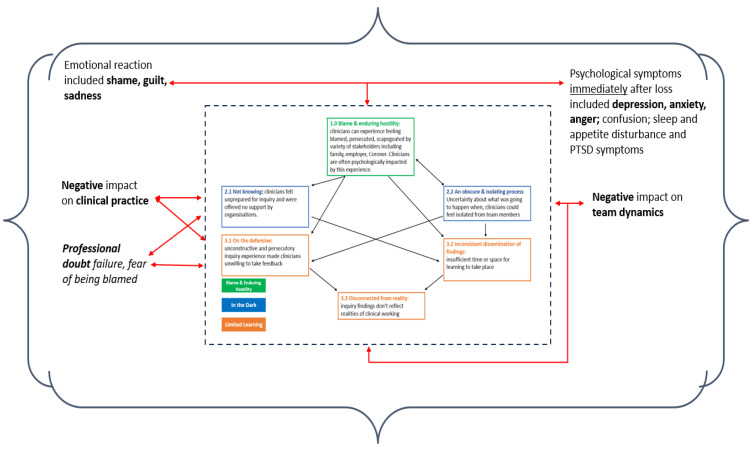
How inquiry processes might inter-relate with the wider experience of losing a patient [27,28,29,30,31,32].

Our data synthesis identified three main themes: Blame and enduring hostility; In the dark; and Limited learning. Themes and sub-themes are shown in Table 4. These themes are inter-related, with one element of a practitioner’s experience impacting another (see Figure 3). The inquiry process is one part of a wider loss experience. How formal inquiry processes might fit into the wider experience of loss is depicted in Figure 4.

Every study included in our review reported that the psychological impact of patient suicide or patient-perpetrated homicide could be exacerbated by the formal inquiry processes that followed. Generally, participants described the process of attending the inquest or inquiry proceedings as stressful and frightening [27,28,30,31]. Occasionally, the feared prospect of attending was later alleviated by a better-than-expected experience [27], but most participants found inquiry processes to be anxiety-inducing.

### 3.3. Blame and Enduring Hostility

A key finding was that participants perceived that formal inquiries operate in a climate of blame [29,31]. Often, this was perceived to be the attitude adopted by those in charge of the inquiry or other stakeholders present including family and media [28,30]. Practitioners described how enduring hostility was an emotionally and physically draining experience [30] and could compound their own sense of guilt and shame associated with the event [27,28,30,32].

Fear of being found at fault worried practitioners irrespective of how experienced they were and was a common source of stress and anxiety [27,31].

“*People go in with a sense of ‘I’m going to be crucified’. It’s an anxiety-provoking episode*”.

Psychiatrist/homicide [31]

The fear could prove reality-based if practitioners felt they were identified as having contributed in some way to the suicide or homicide by their employing organisation, the patient’s family or the coroner [27,28,30,31].

“*The suicide was upsetting, but attending the coroner’s court was traumatic for me. It made me feel like people were out to blame*”.

Consultant Psychiatrist/suicide [28]

Inquiry processes were described as “scapegoating” or “witch hunting exercises” [29], and this appeared to be a common experience, regardless of the type of investigation or where it was taking place. Those leading the inquiry were often perceived to be “insensitive”, “persecutory” or “challenging” in approach [28,30,32].

“*The whole experience was negative, humiliating, criticising*”.

Forensic Psychiatrist(s)/homicide [30]

Whilst most practitioners felt psychologically affected by the death of a patient, the duration of this impact varied. Feeling blamed was frequently cited as a key factor in the ongoing psychological impact of the event [32]. Psychological symptoms experienced by practitioners included depression, anxiety, anger, and appetite loss [27,28,30,32], as well as PTSD-like symptoms [28,30].

“*I was devastated by the incident and subsequent inquiry. I considered leaving medicine early*”.

Consultant Psychiatrist/homicide [32]

In some instances, the process was experienced as fairer than participants had been expecting [27,32], although this was the exception rather than the norm. Inquiries often involve interaction with family members [27,28,30,31], and these could be a further source of anxiety when practitioners experienced family members’ anger being directed at them [27,32]. Sometimes, the prospect of being held accountable prevented practitioners from showing remorse to family members for fear that it would be tantamount to admitting blame [30].

“*The family were very angry and felt their loved one’s death was preventable; they were angry with me personally*”.

Social Worker/suicide [27]

Occasionally, interaction with families was helpful and valued by practitioners [28].

“*The patient’s family were very thoughtful and supportive towards my team even in the midst of their grief*”.

Consultant Psychiatrist/suicide [28]

Despite most practitioners perceiving themselves as being treated punitively, few were referred to a third-party body [30,32], suggesting they were rarely considered to be at fault. This was the case even for practitioners involved in homicide cases where the prospect of legal ramifications is higher. Across the 191 psychiatrists surveyed in the two UK-based studies on patient-perpetrated homicide, six had legal action taken against them and were referred to the General Medical Council (GMC—the medical registration authority for doctors in the UK) [30,32].

## 4. In the Dark

### 4.1. Not Knowing

Participants often described feeling underprepared for the inquiry processes and not knowing what was expected of them. Most surveys asked participants what degree of support they had received after an incident and what support would have been helpful. Feeling more prepared about what to expect from the formal inquiry process was a common suggestion for improving the experience of the process [27,28,30,31].

“*It would help to know what to expect, what about GMC [General Medical Council] referral, what do I do to prepare?*”.

Forensic Psychiatrist/homicide [30]

Some participants described how the information vacuum was filled with secrecy and speculation, fuelling uncertainty and worry.

“*There’s all sorts of other things circulating…that becomes a bit like rumours and gossip because there isn’t a clear process for that [information] to get to those staff*”.

Psychiatrist/homicide [31]

Some participants received no formal support from their employing organisation [32] and felt underprepared for their role in formal processes [27]. Those with less knowledge of the process found the inquiry more emotionally draining than their more experienced counterparts [31]. However, when participants did feel adequately prepared and supported, this could mitigate the stress and fear of the demands of either attending an inquiry or writing a report for it [27].

“*Very good. Interviewed for 3 h […] by trust lawyer the next day, who drafted my statement that I needed for the next 2+ years […] really wise and helpful*”.

Consultant Psychiatrist/homicide [32]

“*My team, manager, clinical director and CEO [chief executive officer] were utterly amazing. CEO called me to check in. Team looked after me. Manager called ahead to a meeting I was chairing to make sure they looked after me*”.

Consultant Psychiatrist/homicide [32]

Suggestions for feeling better prepared included formal education and support from colleagues who had gone through similar experiences [27,30,32].

### 4.2. An Obscure and Isolating Process

Participants frequently found the inquiry process to be obscure and often poorly managed [30].

“*The internal inquiry blamed everyone and was poorly managed. The interview was traumatic, a panel of 8 people, arguing with each other*”.

Forensic Psychiatrist/homicide [30]

Participants involved in homicide cases described how these inquiry processes could last for many years and made it difficult for the practitioner to move on psychologically. Prolonged and uncertain timelines could lead to heightened anxiety and uncertainty [30]. Homicide incidents could involve multiple investigation processes, including the employing organisation, external investigations, public inquiries, criminal proceedings and a coroner’s inquest [30,32].

In both homicide and suicide cases, the impact of a long and complex process, often delayed, could be exacerbated by poor communication from senior management. This increased practitioners’ sense of professional isolation and sometimes impacted negatively on their relationship with their clinical teams by impeding open communication [30,31].

“*There was a time when the team did not even talk to each other, it impacted team cohesiveness*”.

Psychiatrist/homicide [31]

This was especially so in the case of patient-perpetrated homicide inquiries, with individuals feeling deserted by team members wanting to distance themselves from the incident and by the mental health employing organisation appearing to want to protect its reputation [30,32].

“*It made me feel unable to trust colleagues who tried to shift blame from themselves*”.

“*‘It was like a big dysfunctional family… a bird’s nest of bad relationships… where the abused children turn on each other*”.

Forensic Psychiatrist(s)/homicide [30]

In some cases, practitioners intimated they felt purposefully kept in the dark:

“*I learned the meaning of the term Kafka-esque … being prosecuted for something but you don’t know what, and … things around you keep changing in an inexplicable way*”.

Forensic Psychiatrist/homicide [30]

When the converse was experienced, when the inquiry was experienced as fair, with clear and consistent communication [27,31], the fear of attending could be assuaged, but this experience was the exception rather than the norm.

“*harrowing, terrifying, sobering, felt like I was going on trial, but the coroner was clear and made it easier*”.

Nurse/suicide [27]

Better communication and greater levels of transparency in what to expect, were common suggestions for better support [28,30,31].

## 5. Limited Learning

Most participants surveyed across these studies did not find value in inquiry findings. The content of findings was often judged to be irrelevant, whilst the manner in which findings were disseminated precluded the ability of practitioners to reflect usefully on the content.

### 5.1. Inconsistent Dissemination of Findings

The amount of feedback from inquiries and the mode of delivery varied for participants. In Ng et al.’s paper [31], half of the participants reported not having received any feedback at all. When it was given, often there was insufficient time to reflect usefully on it. Some participants reported that the dissemination of feedback was conducted insensitively and in a way that precluded reflection and learning [27]. A more consistent approach to disseminating findings was another suggested area of how support could be improved [31].

“*Maybe there needs to be a greater dialogue between the people doing the inquiry and the service unit in question, to see how things could be framed in a way that was relevant, meaningful [and] achievable*”.

(Psychiatrist)/homicide [31]

### 5.2. Disconnected from Reality

Inquiry findings were described as being hard for practitioners to make sense of owing to a disconnect between findings and the reality of how clinical teams operate [29,31].

“*Too many of them [recommendations] were so poorly worded or generic they didn’t actually make sense as a statement, let alone make sense to the clinical team*”.

Psychiatrist/homicide [31]

Sometimes, the findings were described as focused on individual practitioners rather than systemic weaknesses [27]. In two of the homicide papers [30,31], findings were judged by participants to be of very limited value and often symptomatic of a dysfunctional process where impartiality could not be assured

“*I saw them as biased and unfair*”.

Forensic psychiatrist/homicide [30]

One consultant described being shocked by

“*…the medical member’s punitive approach and the rush to judgment. I also thought that there was some sadistic pleasure in shafting another colleague*”.

Forensic Psychiatrist/homicide [30]

Many participants reflected that the process could be improved through better communication between the different stakeholders. A further suggestion for improved processes was a better separation between clinical and legal considerations. Consultant psychiatrists in Scotland commented that a climate of clinically led learning from adverse incidents would enable constructive analysis but that it should be kept separate from any legal consequences [29].

### 5.3. On the Defensive

The ongoing hostility experienced by many practitioners in relation to the incident (see Theme 1.0) did not appear to be unique to the inquiry process but present before, during and afterwards [27,28,30]. This atmosphere made it difficult for the learning from inquiries to be received constructively [29,32].

“*There is no point after an incident, people coming in and saying we don’t want to blame anyone, we just want to learn. Nobody’s going to buy that. Unless there’s a cultural shift that happens repeatedly with each incident… I don’t think there’ll be much buy-in from the clinical frontline*”.

Psychiatrist/homicide [31]

Learning points were often received defensively, as if acknowledging them might implicate practitioners further [27] and therefore prevented constructive learning [30,31].

“*I felt I had to be defensive*”.

Nurse/suicide [27]

Occasionally, some participants found the process could validate the decisions made [32] with helpful learning outcomes identified, such as completing documentation accurately and advocating for patients [27,31].

“*It confirmed my existing beliefs about the importance of accurate record keeping, including formalising leave cover. It was the hardest thing I have ever dealt with but taught me a lot early on*”.

Practitioner/suicide [27]

“*It’s made me more assertive. When I know my patient needs something, I advocate. If I know they need some sort of support I’d really push for it*”.

Nurse/homicide [31]

## 6. Discussion

The aim of this review was to identify and synthesise what is known about the experiences of mental health practitioners involved in inquiries following a patient suicide or patient-perpetrated homicide. Our research objectives were to identify the potential factors that make inquiry processes more or less difficult, identify the type of support received, and determine whether it was useful. We identified six papers meeting our eligibility criteria. One of these used qualitative interviews and focuse exclusively on the inquiry experience. Five of the studies, all cross-sectional surveys with additional free-text questions, focused on the wider experiences of the practitioner following a patient suicide or patient-perpetrated homicide.

### 6.1. Main Findings of This Review

Inquiries were often experienced as a stressful and anxiety-inducing process. It was very common for participants to either fear being blamed or actively experience it through the attitude of other stakeholders involved in the process. This could include, but was not limited to, coroners, inquiry panel members, family members and the employing organisations. Being blamed appeared to be experienced as a cultural phenomenon rather than a discrete or isolated instance. This was one factor leading to negative psychological consequences for many of the participants across studies.

It was also common for people to feel underprepared for the inquiry process. The amount of formal support given varied. Not knowing what to expect contributed to the anxiety felt by practitioners. Education on what was expected of them and what they could expect from the process were frequently suggested areas for support. Even when sufficiently prepared, the inquiry process could still be experienced as obscure, chaotic and uncertain, with practitioners not knowing what was happening and often feeling professionally isolated. Some practitioners, most often those involved in homicide cases, felt their isolation was a manifestation of being blamed and a cultural need to scapegoat the practitioner.

Inquiry findings were rarely viewed as helpful and could make practitioners feel defensive. Findings were perceived as centering too much on individual action and not enough on systemic weakness. Participants in multiple studies described that the findings and recommendations of inquiries failed to recognise the clinical realities of their work. How findings were disseminated was a further issue, often reported as being conducted in a haphazard or ‘tick box’ way, with limited time for reflection or authentic learning.

Whilst the prevalent experience in the included papers was negative, there were some examples of both suicide and homicide inquiries that were experienced more positively. This tended to be when the reality of attending the inquest was better than feared or when practitioners felt supported by their employing organisation. In these instances, practitioners felt they had been treated fairly. Across these papers, there are select examples of good practices in employing organisations and coroner’s courts. In instances when practitioners felt supported and part of a clear and transparent process [27], the experience appears less likely to be damaging for those involved.

### 6.2. Wider Research

Previously published research in this area is sparse, but findings of the current systematic review can be contextualised within practitioners’ wider experience of patient suicide or patient-perpetrated homicide and by considering the literature on patient family members’ attendance at inquests.

Inquests can be actively harmful to those who attend. This has been evidenced in relation to families [2] and theorised for other stakeholders [5]. The papers in this review suggest that inquests may impact practitioners negatively, especially when internally held feelings of self-blame are, inadvertently or not, reinforced by the practitioner’s employers and in the inquiry processes. The exacerbation of self-blame and professional isolation further undermine the practitioner’s clinical confidence and has been reported to have a negative impact on their mental health and can even lead some clinicians to consider early retirement or to leave the profession [28,30]. However, these papers also report instances when the impact of loss is shorter-lived [27,28,32]. This may suggest that when practitioners do not feel blamed—by themselves or in the investigatory process—and the experience of loss is both recognised and responded to [33], the psychological impact may be less. This is in keeping with Stolorow’s work [34], which suggests that the impact of emotional trauma cannot be explained solely by the pain suffered in the event itself. Rather, the ability to bear the pain is determined by being understood and responded to by others. Thus, responding appropriately to the practitioner’s experience with psychological and practical support may be what allows the pain to be tolerated and processed. More research needs to be conducted to understand this dynamic more fully within this context.

### 6.3. Blame

Feeling blamed after a patient suicide or patient-perpetrated homicide has been written about extensively [16,35,36]. Self-blame has been found to vary between mental health professions. Malik et al. [15] conducted a critical interpretative meta-synthesis of the impact of patient suicide on doctors and nurses and found doctors tended to attribute the death to a failure of (their) doctor-patient relationship, whereas nurses tended to attribute it to a failure of protocol.

The psychological impact of blame can be far-reaching. Self-blame, placing oneself as the protagonist of the story, can be the immediate response of a practitioner following a patient death in a need to make sense of the inexplicable [37]. If this hypothesis holds, the coroner’s inquest, any surrounding inquiry processes and the experience of support received can either validate this narrative or, conversely, challenge it by reconsidering the role the practitioner played versus what they have come to believe. From this vantage point, the internal distress initially felt by practitioners when they hear about the incident can be made better or worse depending on the response of others, such as the employing organisation, colleagues, the patient’s family, and the coroner.

Interviews with consultant psychiatrists after a patient’s suicide or patient-perpetrated homicide explored these experiences by considering the impact on the clinician’s personal, professional, and organisational ‘self’ [3]. In this framework, a patient’s suicide may involve three different forms of loss. The first is a personal bereavement associated with the loss of that patient. The second is a sense of professional loss, often of confidence in clinical decision-making, particularly if the clinician feels they are to blame. The third form is in relation to the clinician’s self within their organisation and team members. Understanding the practitioner’s experience of these inquiries may help us to better comprehend the impact of blame on the practitioner’s ‘organisational’ self. For instance, if the practitioner perceives their value to their team or organisation as altered by the event that has taken place or if they perceive the level of support offered by their employing organisation during the inquiry to reflect their value in some way. It may be that inquiry processes, an intrinsic part of the wider experience of loss, challenge all three parts of the practitioner’s identity, potentially repeatedly.

A systematic review [16] of the impact on mental health practitioners after a patient’s suicide suggested that loss of confidence in clinical decision-making is common. For example, practitioners can become more risk-averse and less comfortable delegating. This can be problematic where the ability to fulfil one’s role effectively is contingent on operating as part of a multi-disciplinary team, as it is for consultants.

### 6.4. Not Knowing

In interviews with bereaved family members attending inquests [2], people often described themselves as part of an investigation process shrouded in obscurity. A combination of not knowing what they could expect the inquest to deliver and operational failings, such as delays or insensitive handling of information, meant the inquest could be chaotic, incapable of meeting expectations, and even prolonging the grieving process for families.

This sense of ‘not knowing’ is echoed in the experience of practitioners identified in this review. Improved support and better communication from the employing organisation may partially alleviate this. However, some practitioners, particularly those involved in homicide cases and subject to lengthier inquiry processes, described a more powerful phenomenon occurring, where they felt they were kept in a state of ‘suspended animation’, which was often experienced as punishing rather than simply frustrating. Practitioners often did not know what to expect next or when the inquiry processes would be over. This compounded the psychological impact of the incident and their sense of professional isolation. When thinking about what support is required, a consistent and clear communication process may prove very effective in helping practitioners challenge their own beliefs when they are stigmatised and professionally isolated. Such simple procedural changes might prove psychologically containing for both the individual and the wider organisation.

### 6.5. Limited Learning

Given the experiences reported, it is unsurprising that practitioners reported that it was difficult to receive inquiry findings constructively. The findings and recommendations were often experienced as critical of them and repeated the experience of feeling poorly supported by the employing organisation. This dynamic is consistent with Stanley and Manthorpe’s [38] observation that in a climate of blame, the ongoing fear of being held responsible prevents meaningful interaction. This may signal a larger cultural challenge of moving beyond a ‘*who dunnit*’ attitude towards one of openness and learning. In this environment, inquiry findings might lead to learning and system-change. In the current climate of blame, inquiry findings – content and delivery – carry two risks: the risk of exacerbating the feelings of blame and isolation repeatedly reported in these studies and the risk of failing to meet demands of change to the public services that are perceived to have failed families. Without these changes, no value can derive from their personal tragedy [39].

### 6.6. Strengths and Limitations of This Paper

To our knowledge, this is the first systematic review that looks at the mental health practitioners’ experiences of attending inquiries after a patient suicide or perpetrated homicide. We conducted a comprehensive search of three databases and searched reference lists of grey literature. We used two independent reviewers for screening, data extraction and quality appraisal. We conducted a thematic synthesis and identified themes prevalent across the underlying studies which help identify research gaps for future study. There were limitations in our approach. We only searched a small number of databases and restricted our papers to English language. Consequently, this review may risk publication bias. Owing to the underlying research base and the types of studies published, our thematic analysis was descriptive with only a limited development of themes. Additionally, owing to the paucity of material directly relevant to our research question, we did not weight our discussion towards higher quality papers. 

### 6.7. Strengths and Limitations of the Included Literature

Within the included literature, there are six papers included in our review, five of which adopted a multi-method approach. The qualitative element of each of these studies enabled us to form an understanding of the practitioner’s experience of inquiry processes after a suicide or homicide and how that experience might differ depending on factors such as support received or the conduct of other stakeholders in the inquiry process. In four of the six studies, there were free-text responses which enabled participants to choose what issues were most meaningful to them without responding to directed questions [40].

Only six papers met the inclusion criteria for this review. Cross-sectional surveys are less empirically robust than other forms of research methods. Our quality assessment suggests that whilst the use of qualitative research is appropriate, none of the papers discussed in detail the research methodologies employed and how this might have impacted the research. As a form of qualitative research, optional, open-ended survey questions are less empirically robust than alternative qualitative methods owing to the risk that answers may be unrepresentative and self-selecting nature, making them less transferable to other study populations [41]. Whilst the papers all had open-ended text responses, some authors chose to report findings but did not include illustrative quotations [29]. Across the six papers, there were 634 participants, 502 of whom were psychiatrists, and 472 of those psychiatrists were consultants, leaving us with limited points of comparison to understand the difference in experience across professional fields and career stages. We also note that three of the six studies [28,30,32] came out of the same body in the Royal College of Psychiatrists. The structure of the three papers is similar, which might overstate the commonality of themes. Finally, there is a risk of response bias. People with negative experiences are more likely to have completed the surveys and shared their experiences.

### 6.8. Areas for Future Research

This area would benefit from more, high quality research to understand the experience and needs of mental health practitioners attending inquests and other inquiry processes.

More research is needed to understand the impact of negative experiences of formal processes on practitioners and the extent to which they influence the mental health practitioners’ own mental health and even potential suicidality. Future research should aim to understand factors within the experience that can make the processes more reparative and constructive for all parties and which of these factors may be addressed through practical support and realistic interventions.

Secondary questions are (1) how the experience of the inquiry process differs between patient suicides and patient-perpetrated homicides and other unexpected deaths, (2) how the experience differs according to the profession, and (3) how the clinician’s personal experience of loss over their lifetimes influence their experiences. These questions will help answer what support is needed for whom.

## 7. Conclusions

Grief after suicide or patient-perpetrated homicide can be complex for those involved in the patient’s care. Practitioners may be required to assist with formal investigations associated with the death. This systematic review identified limited research into practitioners’ experiences of the inquiry process following a patient suicide or patient-perpetrated homicide. The research that has been conducted to date has tended to be in the form of cross-sectional surveys with some ‘free-text’ questions and one small qualitative study. Most of this research has focused on psychiatrists’ experiences, and the experiences of other practitioners are currently not well understood. Whilst acknowledging these limitations, the findings suggest that a) practitioners often experience a sense of blame after the event (both self-blame and from other parties); b) the investigatory process is often experienced as lacking in transparency and persecutory in nature, which exacerbates the negative impact of the event; c) the identified learning outcomes from the investigatory processes are often perceived as unrealistic for implementation in the clinical setting. In addition, the type and usefulness of support received by practitioners after the event varied but was commonly reported as inadequate. The findings suggested some areas that could improve the situation, such as better communication about the inquiry processes and greater support from the employing organisation to prepare for these, but more robust research is needed to inform the nature of these.

## Figures and Tables

**Table 1:** Characteristics and baseline demographics of included studies.

First Author and Publication Year	Country	Topic	Study Design	N	Male (%)	Profession	Yrs Experience	# Events *
Croft, A. 2022 [27]	U.K.	Suicide	Cross sectional survey (including free text responses)	134	19%	53% psychologists and therapists; 33% nurses; 10.5% social workers; 7% support workers; 4% occupational therapists	>10 (68%) 7–10 (10.5%) 4–5 (14%) 1–3 (7%)	1–5 (90%) 10+ (3%)
Gibbons, R. 2019 [28]	U.K.	Suicide	Cross sectional survey (including free text responses)	140	52%	consultant psychiatrists	Not stated	1+ (72%) 6+ (15%) 10+ (3%)
Alexander, D. A. 2000 [29]	U.K.	Suicide	Cross sectional survey (including free text responses)	159	63%	consultant psychiatrists	17.5 mean (SD 17.2)	1 (23%) 2–6 (66%) 7–15 (13%)
Mezey, G. 2021 [30]	U.K.	Homicide	Cross sectional survey (including free text responses) + interviews	26	74%	forensic psychiatrists	>15 (50%) 2–15 (42%) <2 (8%)	1 (70%) 2 (16%) 3+ (12%)
Ng, L. 2021 [31]	New Zealand	Homicide	Semi-structured interviews	10	70%	40% psychiatrists; 40% nursing; 10% social workers; 10% community support workers	2–30 (range)	1 (100%)
Hussain, Q. 2023 [32]	U.K.	Homicide	Cross sectional survey (including free text responses)	165	52%	consultant psychiatrists	21 mean (SD 9)	1 (60%) 2 (16%) Unspecified (39%)

# Events * number of unexpected deaths each participant had experienced.

**Table 2:** Key findings of included studies.

Author (Year), Country	Research Objective	Key Findings
Croft, A. 2022 [27]	To investigate the experiences of mental health practitioners, excluding doctors, following death by suicide and their requirements for support in aftermath	(1) Most reported feeling unprepared for the formal processes that followed the death but felt more knowledgeable having been through it. Prospect of inquest could be frightening but could be mitigated by experiencing a fair and transparent process. (2) Many felt afraid of writing a report for coroner and did not know what to include. (3) Internal inquiry could be insensitively handled with overzealous investigators, inference of blame and poor delivery of feedback. Some did experience good guidance on formal processes and a well-conducted investigation. (4) 72% (82/114) received advice or support for the formal process (55% (63) from their organisation, the rest had informal forms of support). 46% (102) wanted more support for formal processes after a patient suicide.
Gibbons, R. 2019 [28]	To examine how patient suicide affects the emotional well-being and clinical practice of psychiatrists; and the resources that psychiatrists would find helpful before and after the suicide of a patient	(1) Experience of inquest described as ‘very traumatic’, and made some respondents feel upset, sad, angry and that they were being blamed. (2) 19% (15) of respondents commented that the serious incident process was insensitive and/or persecutory. 11% (9) cited coroner’s court as unhelpful with negative factors including the stress of giving evidence; delays; fear of attending and punitive attitude of coroner. (3) 70% (97) of survey respondents asked for support for formal processes following a patient’s suicide. (4) Respondents said the overall experience could be shaped by the attitude adopted by the Trust, the family of the deceased and the coroner.
Alexander, D. A. 2000 [29]	To identify the effect of patients’ suicide on consultant psychiatrists	(1) Fatal Accident Inquiry (*n* = 31) described as unhelpful or very unhelpful by 49%; disciplinary proceedings (*n* = 11) described as unhelpful or very unhelpful by 45%; legal proceedings (*n* = 17) described as unhelpful or very unhelpful by 82%. Conversely, the critical incident review (*n* = 83) described as helpful or very helpful by 78%. 21 of 159 consultants were moderately distressed at prospect of litigation and 12 were extremely distressed. (2) Open text comments suggested clinicians could feel scapegoated, blamed and part of a witch hunt. (3) Formal inquiries could create a climate of blame and impeded a constructive analysis of events. Suggestions for investigation handling: should be clinically led with legal consequences kept separate from critical incident analysis.
Mezey, G. 2021 [30]	To examine the impact on the treating forensic psychiatrist of a patient committing a homicide	(1) Inquiry and legal action ‘one of the most difficult aspects of the overall experience of patient suicide, described as frightening, confusing, punitive and humiliating. (2) Identified issues were length of inquiries, complexity, obscure rules of engagement, highly adversarial and lack of impartiality. These features were present in both internal and external inquiries. (3) No positive aspects of inquiry in terms of learning for clinicians; answers for victim families; righting wrongs or driving improvements.
Ng, L. 2021 [31]	To explore the experiences of clinicians involved with inquiries into the mental health care of patients who were perpetrators of homicide	(1) Inquiry process: most struggled with inquiry interview panel and pointed to disconnect between reality of clinical working and the panel perspective. (2) Varied experiences of dissemination of findings; all wanted more time to reflect on inquiry findings and recommendations. (3) Emotional burden of inquiry included shock at their patient having killed someone; stress at prospect of being sanctioned. (4) Found support through peers, lack of formal support. (5) Detrimental effect on team dynamics made worse by poor communication. (5) Perception of enquiry experience differed across employing Trusts.
Hussain, Q. 2023 [32]	To investigate the experiences and support needs of consultant psychiatrists from all disciplines following a homicide by a patient under their care	(1) 84% (101/121) were involved in an internal inquiry and 2% (4/165) in external disciplinary proceedings. No referrals to GMC. 31% (32/104) provided a report for court and 18% (19) gave evidence. (2) 14% (23/165) felt the formal processes were psychologically damaging, most commonly feeling they were blamed; 5% (9/165) found formal processes constructive and gained valuable experience. (3) 67% (40/60) received no support from their employing organisation and 50% (28/60) said they relied on friends and family. Those who did have support from their employer found it helpful.

**Table 3:** Quality assessment.

			Q1	Q2	1.1	1.2	1.3	1.4	1.5	2.1	2.2	2.3	3.1	3.2	3.3	
Author	Study Design	Rating	Screening		Qualitative					Quantitative			Mixed Methods			MMR Rating
																
Croft, A. 2022 [27]	survey with free text section	64%	Y	Y	Y	Y	Y	N	N	Y	N	Y	Y	Y	N	***
Gibbons, R. 2019 [28]	survey with free text section	57%	Y	Y	Y	Y	N	N	N	Y	N	Y	Y	Y	N	***
Alexander, D. A. 2000 [29]	survey with free text section	64%	Y	Y	Y	Y	N	N	N	Y	Y	Y	Y	Y	N	***
Mezey, G. 2021 [30]	survey + follow up interview	50%	Y	Y	Y	Y	N	N	N	Y	N	N	Y	Y	N	**
Ng, L. 2021 [31]	semi-structured interview	63%	Y	Y	Y	Y	N	Y	N	-	-	-	-	-	-	***
Hussain, Q. 2023 [32]	survey with free text section	64%	Y	Y	Y	Y	N	N	N	Y	Y	Y	Y	Y	N	***

‘Y’ (Yes) = 1; ‘N’ (No) = 0. The quality score for a study = [(sum of ‘yes’ responses/maximum possible score (where all questions answered ‘Y’) × 100. 0–25% *; 26–50% **; 51–75% ***; 76–100% ****. We have excluded questions on the original tool which are not relevant to any of the studies being appraised here. Note that surveys with free text sections or interviews have qualitative and quantitative sections appraised separately in addition to appraisal as a mixed method study [20].

**Table 4:** Themes and sub-themes.

	
1.0 Blame and enduring hostility	
2.0 In the dark	2.1 Not knowing
	2.2 An obscure and isolating process
3.0 Limited learning	3.1 On the defensive
	3.2 Inconsistent dissemination of findings
	3.3 Disconnected from reality

## Supplementary Materials

The following supporting information can be downloaded at: https://www.mdpi.com/article/10.3390/ijerph21030357/s1, Supplementary File S1: Search terms. Full list of search terms (pdf).
