# Supplementary material for: What Are the Experiences of Mental Health Practitioners Involved in a Coroner’s Inquest and Other Inquiry Processes after an Unexpected Death of a Patient? A Systematic Review and Thematic Synthesis of the Literature
Source: Int J Environ Res Public Health. 2024 Mar 18;21(3):357. doi: 10.3390/ijerph21030357 (PMC10970210; doi:10.3390/ijerph21030357)
Supplement: Supplementary file 1 [file ijerph-21-00357-s001.zip › ijerph-2860951-supplementary.pdf]

Supplementary File S1: Search terms

MEDLINE SEARCH STRATEGY
Search Strategy:
----
1 community health workers/ or nursing assistants/ or psychiatric aides/ or nurses, community health/ or occupational therapists/ or general practitioners/ or occupational health physicians/ or psychotherapists/ (23357)
2 *psychiatry/ or *adolescent psychiatry/ or *child psychiatry/ or exp *forensic psychiatry/ or *military psychiatry/ or *psychoanalysis/ or exp *psychology/ or *mental health services/ or emergency services, psychiatric/ or *schizophrenic psychology/ (160862)
3 "Coroners and Medical Examiners"/ (2188)
4 mental health practitioner*.ti,ab. (903)
5 psychiatrist*.ti,ab. (28269)
6 psychologist*.ti,ab. (18617)
7 (mental health adj3 (consultant* or trainee* or personnel* or staff or professional* or counsel*)).ti,ab. (10771)
8 (therapist* or social work*).ti,ab. (62811)
9 or/1-8 (281155)
10 medical errors/ or near miss, healthcare/ (17967)
11 "Root Cause Analysis"/ (418)
12 "Cause of Death"/ (53373)
13 serious investigation*.ti,ab. (58)
14 serious untoward incident*.ti,ab. (16)
15 patient safety event*.ti,ab. (402)
16 patient safety incident*.ti,ab. (603)
17 (safety adj3 inquir*).ti,ab. (71)
18 (incident* adj3 inquir*).ti,ab. (14)
19 (coron* adj3 (inquest* or court* or inquir*)).ti,ab. (334)
20 "root cause analys?s".ti,ab. (1394)
21 or/10-20 (73467)
22 attitude/ or exp "attitude of health personnel"/ or attitude to death/ or exp attitude to health/ or catastrophization/ or optimism/ or pessimism/ or respect/ or stereotyping/ (674058)
23 perception/ or discrimination, psychological/ or perceived discrimination/ or social cognition/ or exp decision making/ or judgment/ (305324)
24 scapegoating/ or psychological distance/ or social stigma/ (15575)
25 (perceive* or perception* or personal satisfaction or perspective* or view* or experience* or need* or issue*).ti,ab. (4800229)
26 (belief* or believe* or opinion* or feel* or knowledge* or understand* or expectation* or attitude* or thought*).ti,ab. (3107277)
27 22 or 23 or 24 or 25 or 26 (7295348)
28 9 and 21 and 27 (523)
29 limit 28 to english language (479)

****
EMBASE SEARCH STRATEGY
Search Strategy:
----
1 mental health care personnel/ (4491)
2 psychiatrist/ (31586)
3 psychologist/ or social worker/ (28801)
4 coroner/ or psychotherapist/ (10233)
5 occupational therapist/ (8106)
6 psychiatric nursing/ or community psychiatric nursing/ (14402)
7 *general practitioner/ (26281)
8 mental health practitioner*.ti,ab. (1094)

9 psychiatrist*.ti,ab. (43252)
10 psychologist*.ti,ab. (28182)
11 (mental health adj3 (consultant* or trainee* or personnel* or staff or professional* or counsel*)).ti,ab. (13303)
12 (therapist* or social work*).ti,ab. (91572)
13 or/1-12 (227740)
14 *"cause of death"/ (12143)
15 "root cause analysis"/ (2789)
16 medical error/ or medical accident/ or "near miss (health care)"/ (20655)
17 therapeutic error/ (1799)
18 serious investigation*.ti,ab. (71)
19 serious untoward incident*.ti,ab. (61)
20 patient safety event*.ti,ab. (527)
21 patient safety incident*.ti,ab. (802)
22 (safety adj3 inquir*).ti,ab. (102)
23 (incident* adj3 inquir*).ti,ab. (22)
24 (coron* adj3 (inquest* or court* or inquir*)).ti,ab. (365)
25 "root cause analys?s".ti,ab. (2849)
26 or/14-25 (39204)
27 *attitude/ or attitude to death/ or attitude to health/ or attitude to illness/ or attitude to life/ or attitude to mental illness/ or employee attitude/ or health personnel attitude/ or optimism/ or pessimism/ or respect/ or risk attitude/ or social worker attitude/ (245860)
28 *perceptive discrimination/ (4470)
29 *stigma/ (5089)
30 social stigma/ (13111)
31 social discrimination/ or employment discrimination/ (5906)
32 clinical decision making/ or ethical decision making/ or medical decision making/ (155569)
33 scapegoating/ (17)
34 (perceive* or perception* or personal satisfaction or perspective* or view* or experience* or need* or issue*).ti,ab. (6239143)
35 (belief* or believe* or opinion* or feel* or knowledge* or understand* or expectation* or attitude* or thought*).ti,ab. (3806712)
36 or/27-35 (8874212)
37 13 and 26 and 36 (438)
38 limit 37 to english language (422)

****

PSYCINFO SEARCH STRATEGY

Search Strategy:

----

1 exp mental health personnel/ (56471)
2 exp social workers/ (14215)
3 counselors/ (9188)
4 therapists/ or occupational therapists/ (14701)
5 exp psychologists/ (34628)
6 *general practitioners/ (4196)
7 mental health practitioner*.ti,ab. (3234)
8 psychiatrist*.ti,ab. (42176)
9 psychologist*.ti,ab. (91512)
10 (mental health adj3 (consultant* or trainee* or personnel* or staff or professional* or counsel*)).ti,ab. (24803)
11 (therapist* or social work*).ti,ab. (135249)
12 or/1-11 (316717)
13 exp causal analysis/ (7556)
14 *patient safety/ (1902)
15 accidents/ (2915)

16 *errors/ or error analysis/ (9594)
17 serious investigation*.ti,ab. (33)
18 serious untoward incident*.ti,ab. (11)
19 patient safety event*.ti,ab. (53)
20 patient safety incident*.ti,ab. (72)
21 (safety adj3 inquir*).ti,ab. (19)
22 (incident* adj3 inquir*).ti,ab. (20)
23 (coron* adj3 (inquest* or court* or inquir*)).ti,ab. (111)
24 "root cause analys?s".ti,ab. (144)
25 or/13-24 (22165)
26 *attitudes/ or counselor attitudes/ or death attitudes/ or employee attitudes/ or employer attitudes/ or health attitudes/ or exp health personnel attitudes/ or occupational attitudes/ or "physical illness (attitudes toward)"/ or psychologist attitudes/ or stereotyped attitudes/ or "work (attitudes toward)"/ (110078)
27 perceptual discrimination/ or social discrimination/ (6192)
28 *stigma/ or mental health stigma/ (12184)
29 *social cognition/ or social categorization/ (13343)
30 *decision making/ (66675)
31 *judgment/ or "clinical judgment (not diagnosis)"/ (24848)
32 (perceive* or perception* or personal satisfaction or perspective* or view* or experience* or need* or issue*).ti,ab. (2162115)
33 (belief* or believe* or opinion* or feel* or knowledge* or understand* or expectation* or attitude* or thought*).ti,ab. (1460682)
34 or/26-33 (2849768)
35 12 and 25 and 34 (466)
36 limit 35 to english language (439)

****

PROQUEST SEARCH STRATEGY

ProQuest CENTRAL (40 results)/SCOPUS (30 results)/WoS (25 results)

"mental health practitioner*" OR psychiatrist* OR psychologist* OR therapist* OR "social work*" OR ("mental health" NEAR/3 (consultant* OR trainee* OR personnel* OR staff OR professional* OR counsel*))

AND

"serious investigation*" OR "serious untoward incident*" OR "patient safety event*" OR "patient safety incident*" OR "root cause analys?s" OR (safety NEAR/3 inquir*) OR (incident* NEAR/3 inquir*) OR (coron* NEAR/3 (inquest* OR court* OR inquir*))

AND

perceive* OR perception* OR "personal satisfaction" OR perspective* OR view* OR experience* OR need* OR issue* OR belief* OR believe* OR opinion* OR feel* OR knowledge* OR understand* OR expectation* OR attitude* OR thought*

English language limit
